# The Dynamics of DNA Methylation in Maize Roots under Pb Stress

**DOI:** 10.3390/ijms151223537

**Published:** 2014-12-17

**Authors:** Haiping Ding, Jian Gao, Cheng Qin, Haixia Ma, Hong Huang, Pan Song, Xirong Luo, Haijian Lin, Ya’ou Shen, Guangtang Pan, Zhiming Zhang

**Affiliations:** 1 Maize Research Institute of Sichuan Agricultural University/Key Laboratory of Biology and Genetic Improvement of Maize in Southwest Region, Ministry of Agriculture, Chengdu 611130, China; E-Mails: dinghp@sicau.edu.cn (H.D.); gaojian8888@gmail.com (J.G.); ma_hai_xia@126.com (H.M.); hhhhsunny@sina.com (H.H.); zhlfan391@163.com (P.S.); linhj521@gmail.com (H.L.); shenyaou@gmail.com (Y.S.); 2 Zunyi Academy of Agricultural Sciences, Zunyi 563102, China; E-Mails: cheng.qin.sicau@gmail.com (C.Q.); luoxirong1982@163.com (X.L.)

**Keywords:** maize (*Zea mays* L.), methylated DNA immunoprecipitation-sequencing (MeDIP-seq), Pb stress, differentially methylated genes (*DMG*)

## Abstract

Plants adapt to adverse conditions through a series of physiological, cellular, and molecular processes, culminating in stress tolerance. However, little is known about the associated regulatory mechanisms at the epigenetic level in maize under lead (Pb) stress. Therefore, in this study, we aimed to compare DNA methylation profiles during the dynamic development of maize roots following Pb treatment to identify candidate genes involved in the response to Pb stress. Methylated DNA immunoprecipitation-sequencing (MeDIP-seq) was used to investigate the genome-wide DNA methylation patterns in maize roots under normal condition (A1) and 3 mM Pb(NO_3_)_2_ stress for 12 h (K2), 24 h (K3) and 48 h (K4). The results showed that the average methylation density was the highest in CpG islands (CGIs), followed by the intergenic regions. Within the gene body, the methylation density of the introns was higher than those of the UTRs and exons. In total, 3857 methylated genes were found in 4 tested samples, including 1805 differentially methylated genes for K2 *versus* A1, 1508 for K3 *versus* A1, and 1660 for K4 *versus* A1. Further analysis showed that 140 genes exhibited altered DNA methylation in all three comparisons, including some well-known stress-responsive transcription factors and proteins, such as MYB, AP2/ERF, bZIP, serine-threonine/tyrosine-proteins, pentatricopeptide repeat proteins, RING zinc finger proteins, F-box proteins, leucine-rich repeat proteins and tetratricopeptide repeat proteins. This study revealed the genome-scale DNA methylation patterns of maize roots in response to Pb exposure and identified candidate genes that potentially regulate root dynamic development under Pb stress at the methylation level.

## 1. Introduction

Plants have developed the ability to adapt to environment changes that adversely affect growth, development and reproduction through various biochemical and physiological processes. Lead (Pb), which is one of the most common pollutants in the environment, readily accumulates in soils and is then absorbed by plants, accumulating in different plant tissues, with the highest amounts generally found in root tissues [1,2]. Some plants have evolved detoxification mechanisms that result in a natural tolerance to heavy metals [3]. These species can accumulate an inordinate amount of heavy metals and inhabited heavy metal-enriched or -contaminated soil, extracting large concentrations of heavy metal Pb into their roots and translocating it to above-ground shoots to produce large quantities of plant biomass [4]. In our previous study, Pb concentrations were measured in the roots and above-ground parts of 19 inbred lines of maize seedlings [5]. Among these lines, line 9782 lacked the ability to hyperaccumulate Pb and showed increased tolerance to Pb stress in the roots and above-ground parts following growth in soil contaminated with 750 mg·kg^−1^ Pb, precluding the threat of Pb entry into the food chain [5]. Moreover, protein catabolic-related genes and transcription factor families, such as bZIP, ERF and GARP, accumulated predominantly in the maize roots during development in response to Pb stress as shown by RNA-seq [6].

Interestingly, maize inbred line 9782 is capable of modulating gene expression in response to Pb stress in a flexible and short-term manner. We speculated that this response occurs via epigenetic modifications, including reversible DNA methylation, histone modifications and chromatin remodeling, and may be associated with hereditable and transgenerational alterations in gene expression [7]. Recently, epigenetic factors, especially DNA methylation, have received considerable attention because of their potential influences on complex traits and responses to adverse environmental conditions, such as drought [8], salt stress [9], and others [10]. Epigenome modifications can occur in stress-related genes through the hypomethylation or hypermethylation of DNA at specific loci or at random loci [11]. In plants, DNA methylation occurs at both symmetric (CpG and CpNpG) and asymmetric (CpNpN) sites through the action of specific *de novo* and maintenance methyltransferases [12]. In recent years, genome-wide analyses of methylation patterns have aided in the detection of differentially methylated regions, and it is probable that these high-throughput methods will provide valuable information in a wide range of fields in plant biology. The genome-wide profiling of DNA methylation levels in different tissues of rice (*Oryza sativa* L.) had few differences in DNA methylation among vegetative tissues compared with those observed between endosperm and other tissues [13]. Robert *et al.* have reported the epigenome-wide inheritance of cytosine methylation variants in a recombinant inbred population in maize [13], and Eichten *et al.* have found epigenetic and genetic influences on DNA methylation variation in maize populations [14]. Nevertheless, the epigenetic mechanisms underlying the response of maize roots to Pb stress remain poorly understood.

The objective of the present study was to use MeDip-seq to assess genome-wide DNA methylation patterns in maize roots and to identify tissues methylated during dynamic development in response to Pb stress using four treatments, including a mock treatment (A1), Pb treatment for 12 h (K2), Pb treatment for 24 h (K3), and Pb treatment for 48 h (K4). The evaluation of the distribution of DNA methylation in the genome of this plant may reveal a large number of differentially methylated genes among these dynamic, responsive time points and identify genes involved in the regulation of the response of maize roots to Pb stress.

## 2. Results

### 2.1. Analysis of Methylated DNA Immunoprecipitation-Sequencing (MeDIP-seq) Reads

**Figure 1:**
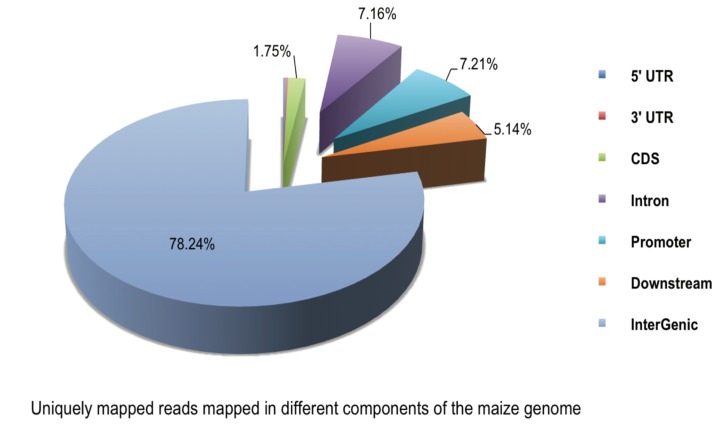
Unique reads mapped in different components of the maize genome (such as promoters, 5' UTRs, 3' UTRs, exons, introns, intergenic regions, CGIs, and downstream regions).

In the present study, four maize roots tissues were used to generate one pooled DNA sample for each group, including a mock-treated group (A1) and those exposed to Pb1000 stress for 12 h (K2), 24 h (K3), and 48 h (K4) (see Experimental Section for details). A range of 6,955,318 to 8,616,236 raw reads was generated for the four groups. More than 80% of the reads were mapped for each group, and approximately 16% of the reads were uniquely mapped to the maize genome. The uniquely mapping reads of A1, K2, K3, and K4 covered 16.13%, 15.95%, 16.27%, and 16.36% of the maize genome, respectively. The proportions of reads uniquely mapped to CpG islands (CGIs) in A1, K2, K3, and K4 were approximately 71.16%, 66.55%, 68.95%, and 63.81%, respectively (Table S1). In addition, analysis of read distributions in different components of the genome showed that the uniquely mapped reads were mainly present in intergenic regions, which contained 80% unique reads, followed by the introns, promoters, and downstream regions. Few reads were mapped to exons, 5' UTRs and 3' UTRs (Figure 1).

### 2.2. DNA Methylation Profiles of Maize Roots

To decipher the genome-wide DNA methylation profiles of the maize roots, we used the uniquely mapped reads to detect the methylated peaks and further analyzed the peak distribution in the different components of the genome through a comparison of methylation densities. We obtained 48,412, 24,599, 34,380, and 38,318 methylated peaks in A1, K2, K3, and K4, respectively (Table S2).

**Figure 2:**
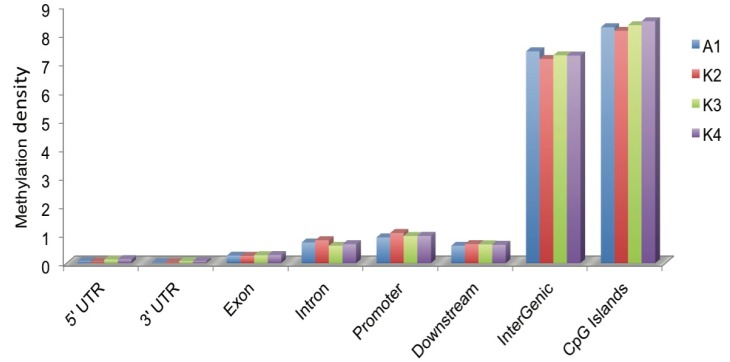
Methylation distributions in different genomic regions. Methylation density within promoter, gene body and intergenic regions was calculated with the ratio of methylated peaks in a particular component to the total area of that region.

The majority of peaks were present in intergenic regions followed by introns and promoters. The comparison of the average methylation densities of the different components of the genome showed that the methylation levels significantly differed (Figure 2). Among all classes, the average methylation density of the intergenic regions was the highest followed by CGIs. The intergenic regions exhibited significantly higher methylation levels than the exon and intron regions (*p* > 0.01). Within the gene body, the methylation density of the introns was significantly higher than those of the UTRs and exons (*p* > 0.01).

### 2.3. Distribution of DNA Methylation in CGIs

It has been reported that CGIs were associated with the majority of the annotated gene promoters. The CGIs were classified into two types based on their methylation statuses. Those containing methylated peaks were regarded as methylated CGIs, and the rest were considered as unmethylated ones. In this study, a total of 356,833 CGIs were scanned by CpGPlot software and detected in the maize genome. Of these, 223,321 were found to be methylated, and 161,777 (72.44%), 153,445 (68.71%), 162,476 (72.75%) and 164,296 CGIs (73.56%) were methylated in A1, K2, K3, and K4, respectively. In addition, most of the methylated CGIs were present in intergenic regions. Within the gene body, the exons showed more methylated CGIs than the UTRs and introns (Table S3). Furthermore, we found that methylated CGIs were enriched in intergenic regions compared with other classes (25%).

### 2.4. Gene Ontology (GO) Analysis of Methylated Genes in the Four Samples

In the present study, the genes that overlapped with the methylation peaks in the promoters or gene body regions were termed as the methylated genes. A total of 223,321 methylated genes were found in the four samples, including 161,777 in A1, 153,445 in K2, 162,476 in K3, and 164,296 in K4 (Figure S1a). Of them, 103,962 were identified in all four groups. Gene ontology (GO) assignments showed that these genes were involved in one or more of the following three categories: biological process, cellular component, and molecular function (Table S4). A total of 33,638 belonged to biological process categories, including cellular process (3519; 10.46%), metabolic process (3880; 11.53%), biological regulation (1310; 3.89%), regulation of biological process (926, 2.75%), response to stimulus (759; 2.26%) and other. Furthermore, 7054 methylated genes belonged to cellular component categories, including cell part (2687; 38.09%), cell (2687; 38.09%), membrane part (576; 8.17%), integral to membrane (476; 6.75%), and extracellular region (147; 2.08%). Additionally, a total of 14,118 methylated genes belonged to be molecular function categories, including catalytic activity (3368, 23.86%), purine nucleoside binding (1120, 7.93%), nucleoside binding (1120, 7.93%), adenyl nucleotide binding (1120, 7.93%) and others (Table S4).

### 2.5. Differentially Methylated Genes among the Four Samples

**Figure 3:**
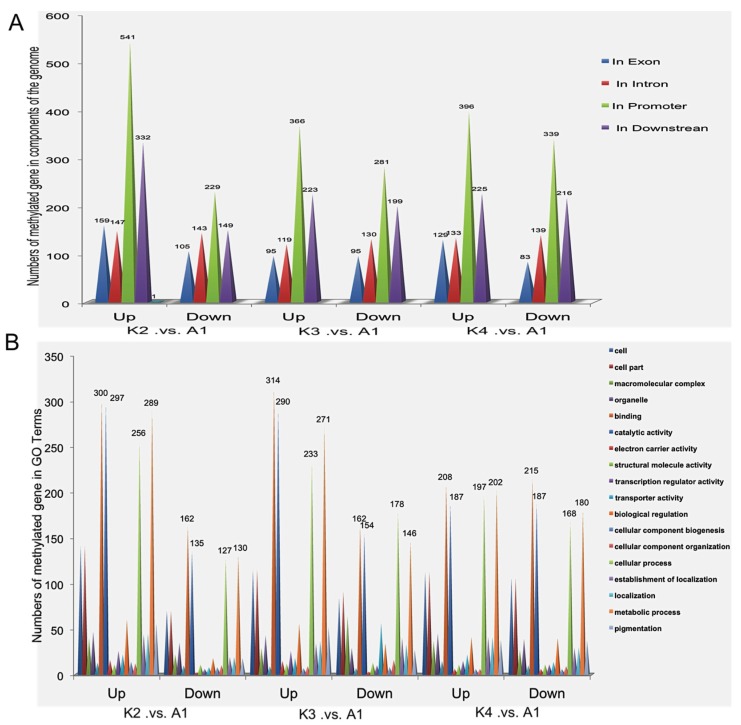
Identification and functional classification of the differentially methylated genes. (**A**) Identification of the differentially methylated genes in components of the genome in all three comparisons, including 1805 differentially methylated genes of K2 *versus* A1, 1508 of K3 *versus* A1, and 1660 of K4 *versus* A1; (**B**) Functional classification of the differentially methylated genes in three comparisons.

Comparison of gene methylation showed that there were 3857 differentially methylated genes (more than two-fold differences, *p* > 0.05) in the four samples, including 1805 differentially methylated genes in K2 *versus* A1, 1508 in K3 *versus* A1, and 1660 in K4 *versus* A1. Moreover, 1170 differentially methylated genes were detected in K2 *versus* A1, and 885 and 1020 differentially methylated genes were identified in K3 *versus* A1 and K4 *versus* A1, respectively (Figure S1b). Of these, 140 genes were differentially methylated in all three comparisons. We subsequently analyzed the direction and degree of methylation difference for the three comparisons in different gene regions. Interestingly, some of transcription factors were found to be associated with Pb treatment, including GRAS, AP2/ERF, bHLH, Myb, ZIF transcription factors. Moreover, some important proteins that might be involved in the response to Pb stress were identified, including serine–threonine/tyrosine-protein kinase, F-box protein, tetratricopeptide repeat protein, ubiquitin, small GTP-binding protein, protein phosphatase 2C (PP2C), plant regulator RWP-RK, glycoside hydrolase family protein, leucine-rich protein and calcium-binding protein (Table 1). Furthermore, the results showed that there were more down-methylated genes than up-methylated genes were accumulated in the K2 *versus* A1, K3 *versus* A1 and K4 *versus* A1, respectively. Most methylated regions with in differentially methylated genes were located in the promoters, followed by the downstream regions, introns and exons (Figure 3A). In addition, GO annotation analysis showed that these genes possessed binding and catalytic functions and were involved in biological regulation, metabolism process and cellular process (Figure 3B). So we speculated that methylation levels were altered following exposure to Pb stress, thereby increasing the low-Pb-responsive differential methylation of genes to cope with the adverse environmental conditions.

**Table 1:** Differentially methylated genes shared by K2 *versus* A1, K3 *versus* A1, and K4 *versus* A1.

Differential Methylated Genes		K2/A1		K3/A1		K4/A1		Interpro_Description	
*GRMZM2G406099*		8.85		10.41		7.01		Tetratricopeptide repeat-containing domain	
*GRMZM2G467695*		0.11		3.54		0.33		Ribosomal protein S4e, central	
*GRMZM2G179910*		0.4		0.46		0.28		Peptidase M17, leucyl aminopeptidase, *C*-terminal	
*GRMZM2G164705*		2.21		2.47		3.63		Mini-chromosome maintenance, DNA-dependent ATPase	
*AC190812.3_FG006*		0.45		0.56		0.51		Small GTP-binding protein domain	
*GRMZM2G398107*		6.32		9.37		7.01		Brevis radix-like domain	
*GRMZM2G480171*		2.64		2.84		2.46		AP2/ERF domain	
*GRMZM2G042133*		0		0.15		0		Universal stress protein A	
*GRMZM2G067320*		4.63		5.2		4.34		Protein phosphatase inhibitor	
*GRMZM2G412577*		0.24		0.4		0.33		Protein of unknown function DUF573	
*GRMZM2G470666*		0.66		2.48		2.23		Peptidyl-prolyl *cis*–*trans* isomerase, FKBP-type, domain	
*GRMZM2G395348*		0.13		0.21		0.1		Unknown	
*GRMZM2G045215*		0		0.12		0		Unknown	
*GRMZM2G406074*		8.85		10.41		7.01		Zinc finger, C_2_H_2_-like	
*GRMZM5G865576*		0.14		2.78		2.29		Zinc finger, C_2_H_2_	
*GRMZM2G065276*		2.53		2.78		2.67		WD4Unknown repeat	
*GRMZM2G035664*		0.38		0.42		0.35		U box domain	
*GRMZM2G010046*		0.25		0.46		0.4		Tify	
*GRMZM2G028114*		1.9		2.01		0		Tetraacyldisaccharide 4'-kinase	
*GRMZM2G007488*		0		1.95		0.36		Small GTP-binding protein domain	
*GRMZM2G139882*		0.36		0.48		0		SANT/Myb domain	
*GRMZM2G134234*		0		0.15		0		Protein of unknown function DUF538	
*GRMZM2G136599*		4.42		4.68		1.6		Unknown	
*GRMZM2G379656*		0.16		0.39		0.25		Unknown	
*GRMZM2G095239*		0.46		0.55		0.44		Zinc finger, RING-type	
*GRMZM2G145458*		3.48		5.2		4.75		Glycosyl transferase, family 48	
*GRMZM2G062499*		4.04		4.37		3.8		F-box domain, cyclin-like	
*GRMZM2G045854*		0.45		0.46		0.31		F-box domain, cyclin-like	
*GRMZM2G057674*		0.2		0.38		0.26		Exocyst complex component Sec1Unknown-like	
*GRMZM2G701221*		0.19		0.32		0.15		CDC48, *N*-terminal subdomain	
*GRMZM2G058292*		0.14		0		0.22		Calponin homology domain	
*GRMZM2G401869*		0.71		0.64		0.79		Unknown	
*GRMZM2G406553*		5.06		5.2		5.51		Unknown	
*GRMZM2G157422*		0.3		0.31		0.27		Unknown	
*GRMZM2G020996*		0.19		0.35		0.19		Unknown	
*GRMZM2G120298*		0		0.09		0.33		Uncharacterised protein family UPFUnknown261	
*GRMZM5G829337*		0.46		0.52		0.58		Protein of unknown function DUF76Unknown	
*GRMZM2G051512*		4.42		5.2		5.51		Protein of unknown function DUF1644	
*GRMZM2G145721*		0.36		0.45		0.48		Plant regulator RWP-RK	
*GRMZM2G008578*		0.45		0.63		0.64		5-Formyltetrahydrofolate cyclo-ligase	
*GRMZM2G315349*		6.32		6.24		10.01		Unknown	
*GRMZM2G108418*		0.59		0.6		0.67		Transcriptional coactivator p15	
*AC202561.3_FG007*		0.24		0.33		10,000		Phosphatidyl serine synthase	
*GRMZM2G121704*		0.46		0.52		0.58		NAD-dependent epimerase/dehydratase	
*AC184857.2_FG006*		3.16		3.64		4.5		LURP1-like domain	
*GRMZM2G145718*		0.36		0.45		0.48		HhH–GPD domain	
*GRMZM2G049950*		0.14		0.69		0.7		Calcium-binding EF-hand	
*GRMZM2G037941*		0.42		0.46		0.53		Unknown	
*GRMZM2G142693*		6.32		7.29		12.01		Unknown	
*GRMZM2G320175*		0.42		0.52		0.58		Pleckstrin homology domain	
*GRMZM2G553532*		0.43		0.46		0.53		Phox-associated domain	
*GRMZM2G103033*		0.47		0.5		0.61		3'–5' exonuclease domain	
*GRMZM2G121820*		7.58		7.29		8.01		Unknown	
*GRMZM2G064814*		0.37		0.37		0.42		Unknown	
*GRMZM2G026442*		0		0.13		1.47		Serine-threonine/tyrosine-protein kinase catalytic domain	
*GRMZM2G066485*		0.47		0.61		0.55		SANT/Myb domain	
*GRMZM2G044900*		0.24		0.33		10,000		Lipase, GDSL	
*AC188195.2_FG004*		0.35		0.35		0.39		Basic-leucine zipper domain	
*GRMZM2G302405*		0.14		0.23		7.01		GRAM	
*GRMZM2G095323*		2.11		2.2		3		Unknown	
*GRMZM2G444567*		10,000		10,000		10,000		K Homology domain, type 1	
*GRMZM2G433216*		0		0		0		Unknown	
*AC213654.3_FG005*		10,000		10,000		10,000		Transcription factor GRAS	
*GRMZM2G133129*		0		0		0		Domain of unknown function DUF292, eukaryotic	
*GRMZM2G089596*		10,000		10,000		10,000		β-Lactamase-like	
*GRMZM5G895991*		10,000		10,000		10,000		Unknown	
*GRMZM2G314946*		10,000		10,000		10,000		Unknown	
*GRMZM2G029055*		10,000		10,000		10,000		Unknown	
*GRMZM2G124524*		10,000		10,000		10,000		Unknown	
*GRMZM2G150866*		10,000		10,000		10,000		Unknown	
*GRMZM2G324886*		10,000		10,000		10,000		UBA-like	
*GRMZM2G471931*		10,000		10,000		10,000		Sec1-like protein	
*GRMZM5G823484*		10,000		10,000		10,000		Proteinase inhibitor I13, potato inhibitor I	
*GRMZM5G845682*		10,000		10,000		10,000		Glycoside hydrolase, family 19, catalytic	
*GRMZM2G080243*		10,000		10,000		10,000		Unknown	
*AC213654.3_FG006*		10,000		10,000		10,000		Ubiquitin interacting motif	
*GRMZM2G031398*		10,000		10,000		10,000		Senescence regulator	
*GRMZM2G159531*		10,000		10,000		10,000		Cytokinin riboside 5'-monophosphate phosphoribohydrolase LOG	
*GRMZM2G025396*		10,000		10,000		10,000		Unknown	
*GRMZM2G017405*		10,000		10,000		10,000		Leucine-rich repeat-containing *N*-terminal, type 2	
*GRMZM2G090213*		10,000		10,000		10,000		FMN-dependent dehydrogenase	
*GRMZM2G132464*		10,000		10,000		10,000		CS-like domain	
*GRMZM2G056524*		10,000		10,000		10,000		Unknown	
*GRMZM2G071277*		10,000		10,000		10,000		Unknown	
*GRMZM2G525084*		10,000		10,000		10,000		Unknown	
*AC229873.1_FG003*		8.85		6.24		7.01		Tetratricopeptide repeat-containing domain	
*GRMZM2G316593*		5.06		4.16		4.5		Rab GDI protein	
*AC190789.3_FG005*		5.06		5.2		5.51		Protein phosphatase 2C (PP2C)-like	
*GRMZM2G380242*		2.18		1.99		2.37		Nucleic acid-binding, OB-fold	
*GRMZM5G862193*		6.32		6.24		10.01		Bromodomain	
*GRMZM2G167718*		3.16		3.12		3.67		Unknown	
*GRMZM2G081380*		2.76		2.46		2.46		Unknown	
*GRMZM2G078389*		0.19		0.16		0.23		Unknown	
*GRMZM2G369243*		0.14		0.12		0.22		Ribosomal RNA adenine methylase transferase	
*GRMZM2G148194*		5.06		4.68		5.51		Protein phosphatase 2C (PP2C)-like	
*GRMZM2G422464*		8.85		6.24		9.01		Mitochondrial carrier protein	
*GRMZM2G057743*		0.49		0.21		6.51		Kinesin, motor domain	
*GRMZM2G385925*		10,000		2.6		10,000		CTLH, *C*-terminal LisH motif	
*GRMZM2G097084*		10,000		0.36		0.4		Aminoacyl-tRNA synthetase, class 1a, anticodon-binding	
*GRMZM2G401075*		7.58		6.24		13.01		Zinc finger, C6HC-type	
*GRMZM2G447876*		0.34		0.33		7.01		Signal recognition particle, SRP9 subunit	
*GRMZM2G427301*		3.16		2.38		3.25		Protein of unknown function DUF5Unknown2	
*GRMZM2G052200*		4.11		3.38		4		Protein of unknown function DUF1754, eukaryotic	
*GRMZM2G093405*		0.55		0.34		0.59		Paraneoplastic encephalomyelitis antigen	
*GRMZM2G392516*		10.11		7.29		8.01		P-type ATPase, A domain	
*GRMZM2G082487*		2.91		2.19		2.4		Leucine-rich repeat	
*GRMZM2G453296*		3.16		0.52		0.53		Knottin	
*AC199487.4_FG002*		2.91		2.39		2.6		Allergen V5/Tpx-1-related	
*GRMZM2G315786*		0.37		0.35		0.38		Zinc finger, RING-type	
*GRMZM2G052880*		10,000		2.08		10,000		WD4Unknown repeat	
*GRMZM2G434669*		7.58		7.29		8.01		Small-subunit processome, Utp11	
*GRMZM2G155260*		3.07		2.38		2.57		Ribosomal protein L2Unknown	
*GRMZM2G061876*		2.37		2.21		2.25		Pentatricopeptide repeat	
*GRMZM2G302233*		0.37		0.26		0.42		Pentatricopeptide repeat	
*GRMZM2G121785*		7.58		7.29		8.01		Pectate lyase/Amb allergen	
*GRMZM2G048883*		5.06		4.16		5.51		Leucine-rich repeat	
*GRMZM2G348780*		5.06		4.16		5.51		Glycoside hydrolase, family 28	
*GRMZM2G035928*		10,000		0.61		0.66		Unknown	
*GRMZM2G037627*		7.58		3.47		4.34		Unknown	
*GRMZM2G072462*		6.32		0.45		9.01		Unknown	
*GRMZM2G395120*		0.44		0.4		2.25		Protein of unknown function DUF159	
*GRMZM5G840726*		4.04		4.37		3.8		WD4Unknown repeat	
*AC184831.3_FG003*		10,000		3.12		3		Kinesin, motor domain	
*GRMZM2G557750*		6.95		4.16		0		Chaperone DnaJ, *C*-terminal	
*AC209877.3_FG002*		2.95		3.12		3.17		Unknown	
*GRMZM2G482657*		5.69		4.16		2.09		Zinc finger, RING-type	
*GRMZM2G003595*		10,000		10,000		1.88		Zinc finger, LSD1-type	
*GRMZM2G075096*		10,000		4.68		4.5		ATPase, AAA-type, conserved site	
*GRMZM2G001904*		2.17		2.03		2		Adenylosuccinate lyase	
*GRMZM2G040079*		6.95		5.72		5		Unknown	
*GRMZM2G011932*		5.06		4.68		4.5		Unknown	
*GRMZM2G152853*		3.48		3.38		3		START domain	
*GRMZM2G702889*		15.17		9.37		8.01		Proteinase inhibitor I13, potato inhibitor I	
*GRMZM2G065205*		10.11		9.37		9.01		Unknown	
*GRMZM2G138410*		10,000		10,000		0.22		Zinc finger, RING-type	
*GRMZM2G040164*		6.95		5.72		5		SANT/Myb domain	
*GRMZM2G435373*		4.42		3.82		0.22		Unknown	
*GRMZM2G339009*		10,000		10,000		0.14		Unknown	
*GRMZM2G348726*		10.11		6.24		2.56		Proteasome, subunit α/β	
*GRMZM2G133958*		10,000		3.12		3		NUDIX hydrolase domain	

### 2.6. Validation of Differentially Methylated Genes by Quantitative Real-Time PCR (qRT-PCR)

**Figure 4:**
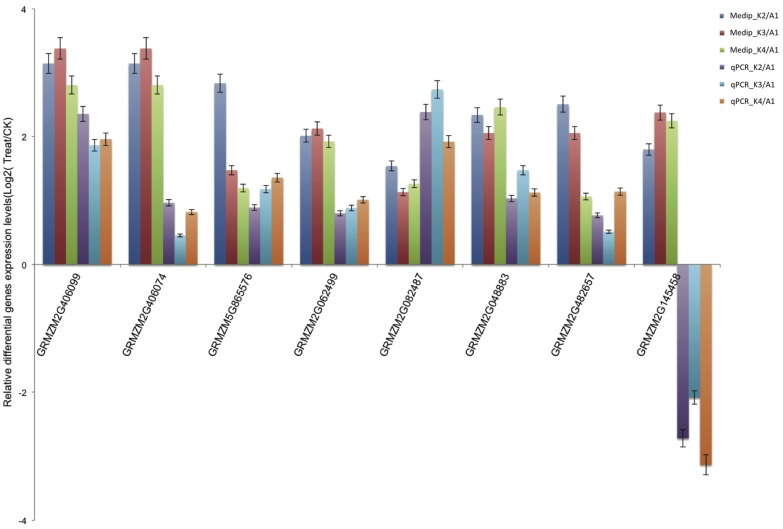
qRT-PCR validation of the Medip-seq data. log2-fold change determined from the relative *C*_t_ values of 8 genes were compared with those detected by Medip-seq. Three replicates for each sample were run and the *C*_t_ values averaged. All *C*_t_ values were normalized to 18s RNA.

To confirm the low-Pb-responsive differentially methylated genes detected by MeDIP-seq, we performed qPCR using three replicates to assess these randomly selected, differentially methylated genes associated with functional categories. The qPCR results showed that these genes were significantly differentially expressed and exhibited contrasting expression compared to the MeDIP-seq data (Figure 4), confirming that these genes were induced under conditions of Pb stress.

### 2.7. Promoter DNA Methylation and Gene Expression Level

**Figure 5:**
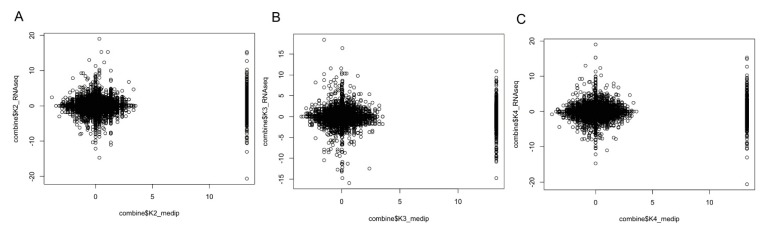
Relationship between DNA methylation in the proximal promoter regions and gene expression level in maize roots responsive to Pb stress for K2 (**A**); K3 (**B**); and K4 (**C**). Genes were assessed according to differential expression levels. DNA methylation level was measured by the log ratio of the *p* value of the methylation peaks, with each point representing the mean expression level and the relative methylation level.

We found that most of the promoter regions were associated with CpG islands and were highly methylated. It is well known that promoter DNA methylation is a repressive signal for gene transcription. We obtained gene differential expression profiles for K2, K3, K4 compared with A1 respectively, using RNA-seq [6]. In the present study, we defined the genomic regions 2 kb upstream and downstream of the gene body as the proximal promoters, and the *p* value of the methylation peaks was used for the methylation level measurements to detect the differentially methylated genes in K2, K3, and K4 compared with A1. We observed that gene expression levels were negatively correlated with DNA methylation in the proximal promoter regions in K2, K3 and K4, whereas there was a relatively lower level of methylation in K3 compared with K2 and K4 (Figure 5).

## 3. Discussion

### 3.1. DNA Methylation Profiles

The characterization of genome-wide patterns of methylation in plant systems has largely been carried out using the model organism *Arabidopsis*. Eichten *et al.* [14] have recently performed methylated DNA immunoprecipitation (ChIP) analysis to locate differentially methylated regions (DMRs) in 51 maize and teosinte inbred genotypes. However, the present study is the first to systematically compare genome-wide maize root methylation profiles in response to Pb stress. Considering that Pb and phosphorus (Pi) would be interact and precipitate in the plant roots, we appropriately increased the concentration of Pi (0.5 mM) by time-course observations of the phenotype and SOD, POD enzyme activity under Pb stress with 3 mM (Figure S2 and Figure S3), which could avoid a Pi deficiency stress. We aimed to identify methylated genes affecting maize roots growth under only heavy metal stress. We used the MeDIP-seq method to investigate genome-wide methylation during dynamic root development in response to Pb treatment. Read distribution analysis found that uniquely mapped reads were enriched in the intergenic regions. In addition, to investigate global methylation patterns, we used Model-based Analysis for ChIP-Seq (MACS) to scan the methylation-enriched regions (called peaks) detected by MeDIP-seq. Peak distribution analysis demonstrated that the promoters were high methylated, whereas the methylation levels in gene body regions were relatively low. Methylation upstream or downstream of genes had repressive effects on gene expression [15,16,17]. Our results support this notion because the promoter-methylated genes had lower expression than those that were not found to be methylated at any component (Figure 5). In addition, the patterns of methylation within and around protein-coding genes were consistent with those observed in previous studies [18,19,20,21]. The 5' and 3' UTRs contained high levels of methylation. Within the transcribed region, methylation was lowest near the transcription start and stop sites and increased away from these sites within the gene body (Figure S4).

### 3.2. Functions of Genes Potentially Methylated in Response to Pb Stress

Plants respond to adverse conditions via a series of physiological, cellular, and molecular processes culminating in stress tolerance. Previous studies have indicated that plants have evolved a range of gene regulatory mechanisms to adapt to different stress responses that act together in various response and defense systems [7]. Transcription factors, transport proteins and some other critical genes are involved in certain signal transduction and secondary metabolite pathways and are considered to be the common stress-related transcripts activated under both biotic and abiotic stresses. In the current study, we found that a total of 140 differentially methylated genes that were identified in all three comparisons (K2 *versus* A1, K3 *versus* A1, and K4 *versus* A1) might contribute to the regulation of the response of maize roots to Pb stress. Among these genes, transcription factors play important regulatory roles in stress responses by regulating their target genes via binding to the cognate *cis*-acting elements [22]. Members of the APETELA2 (AP2), bZIP, NAC, and MYB families have been shown to play regulatory roles in stress responses and have been verified to play significant roles in controlling the expression of specific stress-related genes. In our study, in addition to AP2/ERF, bHLH, MYB, bZIP transcription factors, we also detected the differential methylation of GRAS transcription factor. It has been reported that the salt- and drought-inducible poplar GRAS protein SCL7 confers salt and drought tolerance in *Arabidopsis thaliana* [23]. Knight and Knight (2001) found that the transcription of bZIP, Myb, and zinc finger transcription factor are induced by Pb [24]. In our study, GRMZM2G406099 (AP2/ERF), GRMZM2G048883 (zinc finger, C_2_H_2_-like), GRMZM2G482657 (zinc finger, RING-type) and GRMZM2G062499 (leucine-rich repeat) were validated by qRT-PCR to have decreased methylation levels and thereby increased gene expression levels. It has been demonstrated that plant responses to environmental stresses, including heavy metals, may be regulated by multiple transcription factors. We also found that most of the differentially expressed transcripts were involved in signal transduction and the regulation of gene expression under Pb stress. The first group of genes included those encoding kinases, phosphatases, calcium-binding proteins and proteases that were involved in stress signal transduction. Among them, protein phosphatase (PP) participates in a type of phosphoprotein cascade, resulting in the inactivation of the phosphoprotein. PP has four subunits with different interaction partners for each subunit, whereas PP2B and PP2C are Ca^2+^-dependent. Our results showed that one well-known ABA signal transduction component, GRMZM2G401075 (protein phosphatase 2C (PP2C)-like), was commonly up-regulated [25,26]. F-box protein and U-box protein, which functions as a negative regulator of phytochrome A (phyA)-specific light signaling [27,28], are ubiquitin-related proteins that play important roles in signal transduction in maize during abiotic stress [29]. One gene encoding GRMZM2G406074 (F-box protein) was found and validated in our study. In addition, many tetratricopeptide repeat (TPR) proteins (GRMZM2G082487 and GRMZM2G061876) [30,31] and a ubiquitin-fold modifier 1 (Ufm1) [32] were also identified as commonly up-regulated and directly function as cofactors in Pb stress tolerance without transducing signals. The plant cell wall, which acts as a barrier, plays an important role in regulating heavy metal defense and detoxification by limiting metal uptake and penetration into the protoplast. Many genes involved in cell wall metabolism were found to be repressed in our study. Interestingly, a leucine-rich repeat protein, which has been implicated in cell wall synthesis [33], was present at decreased levels in our study. Two members of the glycoside hydrolase family, GRMZM5G865576 (glycoside hydrolase, family 28) and GRMZM2G145458 (glycosyl transferase, family 48), which decreased in abundance, were identified. These proteins, which are considered to be plasmodesmata-associated [34], are linked to cell elongation and cell wall formation, and the findings herein indicate their involvement in cell wall modification, cell division and growth, enabling a rapid response to Pb stress.

## 4. Experimental Section

### 4.1. Seed Sterilization and Experiment Design

The seeds of maize (*Zea mays*) inbred line 9782 were sown on filter paper saturated with distilled water and incubated at 26 °C in the dark. Three days later, seedlings selected for uniform growth were transplanted into an aerated complete nutrient solution (see Table S5 for details) and maintained for 3 days in a growth chamber with a photoperiod of 14 h light/10 h dark at 26 °C and a relative humidity of 70%. Then, the seedlings were randomly divided to two groups as follows: CK-grown seedlings, which were grown only in half-strength Hoagland solution and Pb1000-grown seedlings, which were grown in CK + Pb1000 (3 mM Pb(NO_3_)_2_) for Pb stress.

### 4.2. DNA Extraction and Preparation for MeDIP-seq

All maize inbred line 9782 root samples were cleaned and immediately frozen in liquid nitrogen for further study. Four libraries were constructed using DNA extracted from the CK-grown (A1) and Pb1000-grown maize roots at 12 h (K2), 24 h (K3) and 48 h (K4) according to the results from POD and SOD assays, respectively [6]. Based on the manufacturer’s protocol, genomic DNA was isolated using a TaKaRa Universal Genomic DNA Extraction Kit Ver. 3.0 (DV811A) (TaKaRa, Osaka, Japan), and then DNA quality was evaluated by agarose gel electrophoresis. DNA samples from three randomly replicated roots within each group were mixed in equal amounts to generate a pooled sample using a Quant-iT dsDNA HS Assay Kit (Invitrogen, Carlsbad, CA, USA). Subsequently, the four pooled-samples were sonicated to produce DNA fragments ranging from 100–500 bp in size. After end repair, phosphorylating and A-tailing with a Paired-End DNA Sample Prep Kit (Illumina, San Diego, CA, USA), the DNA was ligated to an Illumina sequencing primer adaptor. Following the manufacturer’s recommendations, the fragments were used for MeDIP enrichment with a Magnetic Methylated DNA Immunoprecipitation Kit (Diagenod, Liège, Belgium), and the qualifying DNA was used for PCR amplification. Bands between 220 and 320 bp in size were excised from the gel and then purified and with quantified QIAquick Gel Extraction Kit (Qiagen, Valencia, CA, USA) and Quant-iTTM dsDNA HS Assay Kit (Invitrogen, Carlsbad, CA, USA), respectively, on an Agilent 2100 Bioanalyzer (Agilent Technologies, Santa Clara, CA, USA). Following qPCR, DNA libraries were sequenced with an Illumina Hiseq 2000 (Illumina, San Diego, CA, USA) to generate paired-end 50-bp reads by the Beijing Genomics Institute (Bioss, Beijing, China).

### 4.3. Bioinformatic Analysis

First, adapter sequences were removed, and low-quality tags and contamination due to adapter–adapter ligation were filtered out. Next, sequence reads for each tissue were mapped to v2 of the B73 reference pseudomolecules [35] using Bowtie version 0.12.7 [36]. The uniquely mapped data were retained for read distribution analysis, including assessments of the distribution among maize chromosomes and among the different components of the genome. *5b* gene annotation information was downloaded from the maize sequence [35] and the region from transcript start site to transcript end site was defined as gene body region. CpG islands (CGIs) were scanned by CpGPlot [37] with the following criteria: length of 500 bp, GC content of 55%, and observed-to-expected CpG ratio of 0.65. Then, genome-wide methylation peak scanning was conducted using the MACS V 1.4.2 [38,39]. The number of peaks in different components of the maize genome (such as promoters, 5' UTR, 3' UTR, exon, intron, intergenic regions, CGIs, and downstream regions) was analyzed in our study. Moreover, we also analyzed the total peak number in each sample. Overlapping peaks for the different components of the genome were counted as a single peak. The methylation densities of the different components of the genome were compared by calculating the ratio of methylated peaks in a particular component to the total area of that region.

### 4.4. Reverse Transcription, Standard and Real-Time Reverse Transcription PCR

To validate the common differentially methylated genes (DMEs) in the roots as determined by MeDIP-seq, 8 DMEs were subjected to quantitative real-time PCR using an ABI7500 system. *18s* rRNA was used as an endogenous control, and cDNA synthesis was carried out using 1 μg of total RNA. The corresponding primers were designed by Primer5 software and are listed in Table S6. According to the standard ABI7500 system protocol, amplification was performed as follows: 40 cycles of 95 °C for 30 s; 95 °C for 5 s, and 60 °C for 30 s, particularly for the verification of amplification specificity, followed by a thermal denaturing step to generate melting curves. All reactions were run in triplicate, including non-template controls. The threshold cycles (*C*_t_) of each tested gene were averaged for triplicate reactions, and the values were normalized according to the *C*_t_ of the control products of the *18s* rRNA gene. Statistical analysis was performed using the 2^−ΔΔ*C*t^ method.

### 4.5. GO Annotation of All Genes with Peaks

Differentially methylated genes with peaks were used for the subsequent gene ontology (GO) analysis. Genes exhibiting more than 2-fold changes in methylation levels in the different samples were annotated, with a *p* < 0.005 and Benjiamini-adjusted *p* < 0.05, GO functional analysis of the putative target genes was performed by Web Gene Ontology Annotation Plot (WEGO) [40].

## 5. Conclusions

In summary, this study provided a comprehensive analysis of DNA methylation profiles of maize roots and revealed 140 differentially methylated genes that might be involved in response to Pb stress. Our observations provide new clues for elucidating the epigenetic mechanisms of the response of maize to Pb stress, and also provide a foundation for the studies of other types of heavy metal stress in plants.

## Supplementary Materials

Supplementary materials can be found at http://www.mdpi.com/1422-0067/15/12/23537/s1.

## Abbreviations

CGIs: CpG islands
AP2/ERF: APETALA2/ethylene responsive factor
bHLH: basic helix–loop–helix
MYB: myeloblastosis
bZIP: basic leucine zipper domain
GRAS: GAI, RGA, SCR
MeDIP-seq: methylated DNA immunoprecipitation-sequencing
